# Tactical and assessment competency gaps in pre-service coaches: evidence from a game-based implementation

**DOI:** 10.1038/s41598-026-53681-3

**Published:** 2026-05-24

**Authors:** Eyup Acar, Yasin Akinci

**Affiliations:** 1 Department of Physical Education and Sports, Faculty of Sport Sciences, Usak University, Usak, Turkey; 2 Department of Coaching Education, Faculty of Sport Sciences, Usak University, Usak, Turkey

**Keywords:** Tactical competency, Assessment competency, Coach education, Pre-service coaches, Game-based implementation, Mixed methods, Education, Psychology, Psychology

## Abstract

This study examined tactical and assessment competency gaps in pre-service coaches within a structured game-based implementation context. An Explanatory Sequential Mixed Methods design was employed. In the quantitative phase, 40 pre-service candidates (24 Physical Education Teacher Education [PET], 16 Coach Education [CE]) were evaluated using the Educational Game Playing Skill Observation Scale, assessing preparation, implementation, and evaluation competencies. The qualitative phase involved semi-structured interviews with expert academics to provide contextual explanation of the quantitative findings. Results indicated that CE students outperformed PET students in game management and organizational dimensions. However, both groups demonstrated substantial deficiencies in higher-order tactical and assessment competencies, particularly in reflective evaluation, post-game analysis, and in-game tactical facilitation. Effect sizes were in the small-to-medium range yet consistently indicated structural competency gaps extending beyond technical proficiency. These findings suggest that current coach education pathways insufficiently develop tactical facilitation and evaluative expertise within game-based instructional practices. Systematic curricular integration of practice-based modules explicitly targeting tactical intervention and performance evaluation is recommended.

**Supplementary Information:**

The online version contains supplementary material available at 10.1038/s41598-026-53681-3.

## Introduction

Physical education and sport pedagogy does not view an individual’s participation in physical activity as limited only to motor skill acquisition, but rather as a multi-dimensional process that also encompasses cognitive, affective, and social development domains^1^. In line with this approach, it is advocated that game-based learning (GBL) environments and their structured implementation should gain a central place in educational programmes because they support learning holistically^2,3^. Educational games, as structured activities for learning purposes with specific objectives, rules, and cognitive demands, carry the potential to develop participants’ higher-order skills such as decision-making, problem-solving, collaboration, and self-regulation^4,5^.

This pedagogical potential necessitates that games in instructional processes are evaluated not only as enjoyable tools that increase physical activity but also as contextual carriers of deep learning^6,7^. Indeed, game-centred models such as Teaching Games for Understanding (TGfU)^8^, Game Sense^9^, and similar approaches aim to transform instruction from a linear process focused on technical skill transfer to an understanding built upon in-game decision-making, tactical awareness, and problem-solving^10^. These approaches reveal that the educator’s game playing competency is not merely a physical organisation skill, but involves multi-layered components such as coaching knowledge, learning design, and the assessment of learning outcomes^11^.

The pedagogical value of educational games is supported by fundamental theoretical frameworks relevant to both Coach Education (CE) and Physical Education Teacher (PET) education. In the context of coaching, educational games are central to approaches such as Long-Term Athletic Development (LTAD)^12^ or the Developmental Sport Participation Model (DSPM)^13^. These frameworks, while primarily targeting the long-term development of athletes, are crucial for pre-service coaches as they provide the foundational understanding for designing and implementing effective game-based instruction. The study focuses on how pre-service coaches and teachers are prepared to apply these models, rather than on the models themselves. These models transform the coach’s role into a facilitator who designs the game environment, observes, and guides the athlete with correct questions at critical moments, aiming to increase athletes’ game intelligence and tactical awareness^5,9^. Due to the nature of CE, the LTAD/DSPM framework prioritises an intervention skill focused on performance and outcome; this theoretically supports pre-service coaches exhibiting higher proficiency in sub-dimensions such as game management and flow control by enabling them to be more directive, rapid, and outcome-oriented in their in-game interventions. In the field of PET Education, educational games aim to support the comprehensive participation of students and lifelong physical activity skills through tactical approaches like TGfU and Game Sense, as well as principles of Cooperative Learning^14^ and the Sport Education model^15^. TGfU’s core emphasis on tactical decision-making and student-centred inquiry is a more complex process requiring pedagogical reflection compared to the outcome-focused intervention of CE. For both professional fields, the success of educational games is directly dependent on the educator’s game playing competence; that is, their ability to design, manage, stop, ask critical questions, and evaluate the game.

The success of game-based approaches (e.g., TGfU, Game Sense) is directly contingent upon the educator’s game playing competency, which involves not only the transmission of technical skills but also the ability to optimize the game environment for athlete development^9,16^. In this context, the sports science literature utilizes the concept of Coaching Content Knowledge (CCK) to define the coach’s knowledge base^17^. CCK integrates elements such as the coach’s subject matter knowledge (rules, techniques), pedagogical knowledge (instructional strategies), and context knowledge (athletes’ developmental stages). Tactical Competency emerges as a critical application of this CCK, referring to the coach’s skill in reading the flow of the game, understanding athletes’ decision-making processes, and making timely, appropriate interventions to enhance performance^5^. Our current study specifically focuses on the deficiencies in Tactical Competency demonstrated by CE students within game-based learning environments. Fundamental coaching frameworks like the LTAD^12^ and the DSPM^13^ have transformed the coach’s role into a facilitator who designs the environment to enhance the athlete’s game intelligence and tactical awareness^5,9^. The effectiveness of these models relies heavily on the coach’s ability to stop the game, ask critical questions, and reorganize the game to develop the athlete’s decision-making skills, which is essentially their Tactical Competency. Therefore, the systematic evaluation and development of this competency in CE curricula are vital for the continuity of athlete performance and development.

Although numerous studies have been conducted in the literature on the effects of game-based models on student outcomes^18–20^, studies focusing on how pre-service educators implement and operationalize game-based learning principles in authentic instructional contexts remain limited^21^. Competent game playing should be considered a multi-layered proficiency, extending from planning to in-game interventions, evaluation, and feedback^14,22^. However, it is insufficiently known how such skills are acquired in current teacher and CE programmes, at what level they develop, and in which components deficiencies exist^11,22,23^. This deficiency reveals a situation where the competencies of pre-service educators in designing and managing educational games for appropriate purposes are not systematically evaluated, and consequently, educational interventions targeting these skills cannot be structured based on data.

Despite the established theoretical importance of game-centred pedagogies like TGfU^8^ and LTAD^12^, a critical gap persists in the literature regarding the application competence of pre-service educators. While existing research often focuses on student outcomes, there is a profound lack of systematic, mixed-methods evaluation of the educator’s ability to transition from technical game management to tactical game facilitation. Specifically, the literature has failed to adequately address (a) the comparative differences in game playing competencies between pre-service teachers and coaches considering their distinct professional philosophies, and (b) the extent to which critical skills, such as tactical intervention, post-game evaluation, and reflective practice, are developed in current training programs. This deficiency is not merely a methodological oversight; it represents a failure to empirically assess the CCK necessary for the effective implementation of game-based learning. This critical gap in empirical assessment of CCK directly informs the necessity of the present study, which seeks to evaluate these competencies within a structured game-based implementation context.

A critical distinction in the application of game-based models lies in the professional trajectory and pedagogical socialization of the practitioner. While in-service coaches often rely on intuitive, experience-based “craft knowledge” to navigate the complexities of game-based environments, pre-service coaches are frequently caught in a “theory-practice gap,” where they possess theoretical understanding but struggle with the fluid, non-linear demands of authentic implementation^24,25^. This divergence highlights the importance of understanding the distinct pathways of coach and teacher education, a topic extensively explored by scholars such as Cushion and Cope in their foundational work on CE^26^, and more recently by Demiral and Naziroglu in their comparative analysis of pedagogical approaches^27^. These studies underscore that the preparation of pre-service educators is not monolithic; rather, it is shaped by the specific professional roles they are being trained for, leading to differential emphasis on pedagogical content knowledge, tactical understanding, and reflective practice. This study specifically targets pre-service coaches to examine the foundational development of these competencies before they are solidified through years of potentially unreflective practice. Furthermore, the consistency of training systems for pre-service educators is heavily influenced by localized educational policies and cultural contexts. In the Turkish higher education system, a notable paradox exists: PET candidates are admitted to programs based on significantly higher national university entrance exam scores, reflecting superior academic standing compared to CE candidates^28,29^. However, the CE curriculum is characterized by a more intensive integration of theory and practice through “Specialization Courses”. These modules explicitly incorporate Long-Term Athletic Development (LTAD) models, providing detailed training on age-appropriate exercise content and the psychological, cognitive, and emotional development of children through practical application and creative design tasks^30^. In contrast, PET curricula, while academically rigorous, often lack the same level of sustained, performance-oriented practical immersion^31^. This unique localized context provides a compelling rationale for comparing these two cohorts, as it allows for an investigation into whether specialized, practice-heavy curricula (CE) can compensate for lower initial academic entry scores in the development of complex tactical and assessment competencies.

The main objective of this study is to evaluate how pre-service educators implement game-based learning principles through observable tactical and assessment practices. The main rationale for the study is to examine the potential differences in educational game playing competencies between two candidate groups with different professional focuses (teaching and coaching) in light of the theoretical frameworks mentioned above. Mixed-methods research combining qualitative and quantitative methods offers the opportunity to multi-dimensionally assess the practice-based competencies of pre-service teachers^32^. Specifically, systematic evaluations conducted by expert observers can objectively reveal which areas of these skills are strong and which areas are open to development. In this sense, the study examines competency gaps emerging within structured game-based implementation practices rather than isolated technical performance tasks.

### Theoretical framework and hypothesis development

The formulation of the research hypotheses is grounded in the intersection of CCK and Occupational Socialization Theory (OST). CCK, as defined by Cushion, encompasses the coach’s subject matter knowledge, pedagogical knowledge, and context knowledge^17^. OST, particularly Lortie’s concept of the “apprenticeship of observation,” posits that individuals entering a profession are heavily influenced by their prolonged exposure to that profession as recipients of its services^33^. In the context of coaching and teaching, this implies that pre-service educators’ perceptions and approaches to game-based instruction are shaped not only by formal training but also by years of observing coaches and teachers. This theoretical lens allows us to hypothesize differential development of competencies based on the distinct socialization pathways of CE and PET students.

Accordingly, the research tests the following hypotheses in light of the theoretical justifications outlined above:

- H_1_: Pre-service coaches enrolled in the CE department are expected to have significantly higher total Educational Game Playing Skill scores compared to pre-service teachers enrolled in the PET Education department.
- H_2_: Pre-service coaches enrolled in the CE department are expected to have significantly higher scores in the sub-dimensions of Educational Game Playing Skills that require directive intervention, such as game management and flow control, compared to pre-service teachers enrolled in the PET Education department.
- H_3_: Qualitative interviews with expert academics will support the quantitative findings, revealing that the areas requiring development for pre-service coaches (especially tactical decision-making and reflective evaluation required by CCK) will be indicated more frequently compared to pre-service teachers enrolled in the PET Education department.

Our study addresses this critical void by employing a robust Explanatory Sequential Mixed Methods design to provide an in-depth, evidence-based analysis of these competencies. The findings are intended to inform a global paradigm shift in the curriculum design of PET and CE programs. Structured within the framework of these hypotheses, the study is conducted to restructure the educational game-based instruction dimensions of teacher and CE programmes and to develop practice-oriented recommendations.

## Method

### Research design

This study was designed within the scope of a “Mixed Methods Research” approach to multi-dimensionally evaluate the coaching competencies of pre-service educators. In the qualitative follow-up phase, expert academics participated in semi-structured interviews guided by a study-specific interview form, the full English version of which is presented in Supplementary File 2 (Interview Guide). The “Explanatory Sequential Design” was adopted in the research, where quantitative data were collected in the initial phase; subsequently, these findings were supported by qualitative data to provide an in-depth contextual explanation^32^.

In this context, the research was conducted in two sequential phases:

*Quantitative Phase*: The educational game playing competencies of the pre-service educators were evaluated using a standardised measurement tool through the scoring of expert observers. *Qualitative Phase*: Semi-structured interviews were conducted with expert observers to explain and interpret the quantitative findings. This phase was based on the principle that qualitative data serve an explanatory function.

### Participants

The study sample consisted of 40 pre-service educators (PET: *n* = 24; CE: *n* = 16) enrolled in a prominent sports science faculty in Turkey. The participants’ ages ranged from 19 to 26 years (M_age_} = 21.45, SD = 1.62). Gender distribution was relatively balanced, with 35% female (*n* = 14) and 65% female (*n* = 26) participants. While all participants were “pre-service” (i.e., had not yet entered formal full-time employment), their prior sports experience varied; CE candidates reported an average of 6.2 years (SD = 2.1) of competitive sports participation, while PET candidates reported 4.8 years (SD = 2.4). All participants were in their final two years of their respective four-year undergraduate programs, ensuring they had completed core pedagogical and sport-specific coursework. For the qualitative phase, expert academics were purposively selected, not students. This decision was critical to the explanatory sequential mixed-methods design, where the qualitative data aimed to provide a deeper, theoretically grounded interpretation of the quantitative findings. Expert academics, with their extensive pedagogical and research experience in game-based learning, were uniquely positioned to offer nuanced insights into the observed competency gaps and contextual factors influencing pre-service educators’ performance. Their role was not to reflect on their own pre-service experiences, but to analyse and interpret the performance of the pre-service coaches and teachers, thereby providing an external, informed perspective that students could not offer.

### Program comparison

To minimize bias in the comparative analysis and clarify the structural differences between the two cohorts, a summary of the training programs is provided in Table 1. (Table 1 here)

**Table 1:** Structural and Curricular Comparison of PET and CE Programs in the Local Context.

Feature	PET	CE
Admission Criteria	High national university entrance exam scores (Top 10–15% percentile).	Moderate national university entrance exam scores.
Primary Focus	Broad pedagogical knowledge, school-based PE curriculum, and multi-sport literacy.	Sport-specific performance, tactical management, and athlete development.
Curriculum Structure	Emphasis on educational psychology, sociology, and general teaching methods.	Emphasis on “Specialization Courses” in a specific sport branch.
LTAD Integration	General exposure to developmental stages in PE contexts.	Intensive, multi-semester training on Long-Term Athletic Development (LTAD) models.
Practical Immersion	School-based internships focusing on classroom management.	Practice-based modules focusing on tactical intervention and performance evaluation.
Target Population	K-12 students in general education settings.	Athletes across the developmental spectrum (recreational to competitive).

### Expert characteristics

The expert evaluation group comprised five academics specialised in sport pedagogy and educational games at national and international levels. The following objective criteria were observed in the selection of experts: Having published in peer-reviewed journals on educational games, game-based learning, and pedagogical models (TGfU, Game Sense, etc.), having at least 10 years of academic experience, holding at least a PhD degree, and having previously taught a course based on educational games in at least one teacher education programme. Two of these experts participated in the quantitative phase (scoring) of the research, while all five experts participated in the qualitative phase (interview). Although the sample size was modest, medium effect sizes and high inter-rater reliability coefficients strengthen the internal validity of the findings. Moreover, the explanatory sequential mixed-methods design enhances analytical depth beyond purely statistical inference.

### Ethical compliance

The research data were collected in accordance with the permission (04-04-2024/2024-87) obtained from the Usak University Scientific Research and Publication Ethics Committee for Social and Human Sciences. Informed consent was obtained from the participants before their involvement in the study. All processes adhered to the ethical principles of the Declaration of Helsinki, participation was voluntary, and the confidentiality of personal data was protected^34^.

### Data collection instruments

#### Educational game playing skill observation scale

In the quantitative phase of the research, the Educational Game Playing Skill Observation Scale developed by^35^ was used to measure the coaching competencies of the pre-service educators during the game playing process. This scale consists of three sub-dimensions: Preparation Phase (5 items), Game Phase (19 items), and Evaluation Phase (4 items). Each item was scored on a 4-point Likert-type scale (1 = Not at all appropriate, 4 = Very appropriate). The total score of the scale ranges from 30 to 120. The internal consistency of the scale in this study was calculated as Cronbach’s alpha = 0.92, which indicates a high level of reliability. (Table 2 here)

**Table 2:** Representative items from each sub dimensions of the educational game playing skill observation scale.

Sub-dimension	Representative item	Corresponding coaching skill
Preparation Phase	“Prepared the game area and materials appropriately in advance”	Organizational CCK
Game Phase	“Makes timely interventions in the game (diversifying, balancing the game, reorganizing, etc.)”	Tactical Intervention CCK (Instructional Strategies Knowledge)
Evaluation Phase	“ Conducted evaluations appropriate to the game’s objective.”	Reflective CCK (Knowledge of Learning Outcomes and Assessment)

The scale was selected due to its strong alignment with the core principles of game-centred approaches, particularly its focus on the educator’s role in facilitating tactical decision-making and post-game reflection, thereby ensuring high content validity for assessing TGfU-related competencies. The scale demonstrates strong alignment with game-cantered pedagogical frameworks, particularly in operationalizing tactical facilitation and reflective evaluation behaviours.

To enhance methodological transparency and allow for a deeper understanding of the observed constructs, Table 2 presents representative items from each sub-dimension of the scale (The full scale is available in the Supplementary Material 1). These examples demonstrate specific behaviours that expert observers are tasked with evaluating.

#### Consistency of expert evaluations

Cohen’s kappa coefficient was used to test the reliability of the experts’ observation scores. Cohen’s kappa coefficient is a statistical measure of inter-rater agreement for qualitative items^36^. Pearson’s R values and 95% confidence interval measurements were also tested (See. Table 3).

**Table 3:** Consistency of expert evaluations.

Sub-dimension	Kappa	Pearson’s *R*	%95 CI	Approximate T^b^	Sig.
Preparation Phase	0.86	0.88	[0.84 – 0.90]	13.03	< 0.001
Game Implementation Phase	0.85	0.88	[0.84 – 0.90]	13.53	< 0.001
Evaluation Phase	0.91	0.95	[0.93 – 0.96]	18.73	< 0.001
Total Score	0.90	0.91	[0.88 – 0.93]	13.81	< 0.001

High kappa coefficients (0.85–0.91) indicate ‘almost perfect’ agreement between expert assessments^37^. Similarly, the high level of Pearson correlation coefficients (0.88–0.95) supports a strong linear relationship between experts in terms of continuous scoring. Furthermore, the high (13.03–18.73) and significant (*p* < .001) approximate T values across all subscales indicate that observer agreement is not random and is statistically very strong. Taken together, these results suggest that expert ratings are highly reliable and repeatable in both categorical and continuous measurements.

#### Semi-structured interview form

The semi-structured interview form used to collect qualitative data was developed specifically for this study by the research team and consisted of four open-ended questions aligned with the three core sub-dimensions of the Educational Game Playing Skill Observation Scale.

The complete English version of the interview guide is provided in Supplementary File 2 (Interview Guide) to ensure full transparency and replicability.

Content validity was established through consultation with two independent experts, who achieved consensus on the questions’ clarity, relevance to the research objectives, and non-overlapping content coverage. The form was specifically designed to enable expert observers who had previously evaluated the candidates’ video-recorded educational game applications to provide pedagogically grounded interpretations of observed strengths and weaknesses across all three phases of the game process.

### Data collection procedure

The game-based implementations were structured classroom applications designed in accordance with game-centered pedagogical principles (e.g., TGfU, LTAD-informed practices), ensuring ecological validity. The implementation process of the research was carried out in two stages:

#### Quantitative stage

A total of 40 educational game applications conducted by the pre-service educators were documented via video recording in a classroom setting. Each video was independently and blindly reviewed and assessed by two experts. The videos were presented to the experts in random order without any identifying information. Each expert worked individually, evaluating a maximum of 10 videos per day to reduce evaluation fatigue and increase scoring reliability. The experts worked separately, and scores were not shared on a common platform.

#### Qualitative stage

Following the quantitative analyses, individual semi-structured interviews were conducted with the experts. These interviews were conducted using a structured set of prompts derived from the study-specific interview guide, the full English version of which is available in Supplementary File 2 (Interview Guide). In these interviews, questions such as “What kind of evaluation process did the candidate conduct with the students regarding learning outcomes after the game? Could they establish a relationship with the targeted gains?” and “How would you evaluate the candidate’s process of planning the educational game, arranging materials, and explaining the rules? Could you specify the aspects you found strong or insufficient?” were used to deepen the pedagogical and contextual explanation of the findings. The interviews lasted an average of 25–40 min, audio recordings were taken and then transcribed verbatim.

### Data analysis

#### Quantitative data analysis

The quantitative data were analysed using the IBM SPSS Statistics 26.0 software. Descriptive statistics (mean, standard deviation) were calculated; an independent samples t-test was applied to examine the differences between the two departments in educational game playing scores^38^. Crucially, Cohen’s d values were calculated and reported for all significant differences to determine the practical significance (effect size) of the findings, with the magnitude of the effects interpreted according to established guidelines^39^.

#### Qualitative data analysis

The qualitative data were analysed using the thematic analysis method^40^. All interview transcripts were generated from audio-recorded sessions conducted with the experts using the semi-structured interview guide detailed in Supplementary File 2 (Interview Guide). An overview of the finalized coding structure is presented in Table 4. The coding process was carried out independently by two researchers; consensus was reached on the themes. Inter-coder agreement was calculated with Cohen’s Kappa (κ = 0.79, *p* < .001) coefficient, this value indicates a high level of coding reliability^38^. A detailed description of the thematic analysis process, including the initial coding, theme development, and final theme definitions, is provided in the Supplementary Material to ensure maximum methodological transparency.

**Table 4:** Coding scheme: educational game playing process themes.

Main theme	Sub-theme	Description
1. Preparation and Organisation	1.1 Material Planning	Arrangement of space, equipment, and player setup
	1.2 Clarity of Rules	Delivery of simple, clear, and age-appropriate game rules
	1.3 Safety and Risk Management	Prioritisation of student safety during the session
2. In-Game Implementation	2.1 Leadership and Communication	Classroom management with an energetic and motivational stance
	2.2 Intervention Strategies	Ability to pause, guide, and restart the game as needed
	2.3 Adaptability and Flexibility	Modifying rules or structure in response to the flow of the game
3. Tactical Instruction	3.1 In-Game Questioning	Use of open-ended questions to promote tactical thinking
	3.2 Decision-Making Support	Providing opportunities for players to make decisions during gameplay
4. Evaluation and Reflection	4.1 Linking Game to Pedagogical Goals	Highlighting learning outcomes at the end of the game
	4.2 Form of Feedback	Providing structured feedback at both individual and group levels
5. Professional Reflection	5.1 Self-Evaluation of Practice	Candidate’s level of awareness regarding their own instructional performance
	5.2 Insight into Developmental Needs	Candidate’s ability to identify personal strengths and areas for improvement

### Data integration

Data integration was performed sequentially and complementarily, in accordance with the principles of the explanatory sequential design. First, the findings obtained from the quantitative phase were used to guide the thematic coding; subsequently, the quantitative results were interpreted in a pedagogically explanatory follow-up manner through the qualitative findings. This strategy allowed the findings to be interpreted not only statistically but also pedagogically.

## Results

The quantitative and qualitative findings obtained from the expert evaluations regarding the educational game playing competencies of pre-service educators are presented in this section with a holistic approach. The findings are structured in accordance with the explanatory sequential mixed-methods design of the research; first, the quantitative findings based on expert scoring are presented, followed by the qualitative themes that support this data.

### Quantitative findings: general competency levels

The game playing competencies of the 40 pre-service educators (24 PET Education, 16 CE) were independently evaluated by five expert academics. The general findings indicate that the overall game playing competencies of the pre-service educators are at a sufficient level (M = 3.22, SD = 0.39). Specifically, the “Preparation Phase” received the highest average score, while the “Evaluation Phase” stood out as a relatively low-performance area. This situation reveals that the candidates are strong in organization and planning skills before the application but require more development in managing the pedagogical evaluation processes after the game. Descriptive statistics for the overall competency levels are presented in Table 5.

**Table 5:** Descriptive statistics for educational game playing skill levels.

Sub-dimension	*N* of Items	M	SD	Skill Level
Preparation Phase	5	3.32	0.43	Very Good
Game Implementation	21	3.23	0.44	Sufficient
Evaluation Phase	4	3.03	0.55	Sufficient
Overall Competency	30	3.22	0.39	Sufficient

Scale score ranges: 2.51–3.25: Sufficient; 3.26–4.00: Very Good.

### Quantitative comparisons: differences between departments

An independent samples t-test was conducted to compare the Educational Game Playing Skill scores between CE and PET students. As hypothesized, CE students obtained statistically significantly higher scores than PET Education students across all sub-dimensions. The results, including the effect size (Cohen’s d), are presented in Table 6.

**Table 6:** Comparison of Educational Game Playing Skill Scores Between CE and PET Students.

Sub-Dimension	Group	*N*	X̄	SD	t(38)	*p*	Cohen’s d
Preparation Phase	CE	16	3,43	0.32	4.01	< 0.001	0.38 (Small)
	PET	24	3.27	0.46			
Game Implementation	CE	16	3.41	0.42	6.37	< 0.001	0.67 (Medium)
	PET	24	3.13	0.41			
Evaluation Phase	CE	16	3.23	0.35	6.35	< 0.001	0.58 (Medium)
	PET	24	2.93	0.61			
Overall Competency	CE	16	3.39	0.34	6.94	< 0.001	0.70 (Medium)
	PET	24	3.13	0.39			

*N* = 400 represents the total number of observation points derived from the assessment of 40 participants across5 distinct evaluation criteria of the 2 experts within the Educational Game Playing Skill Observation Scale.

The statistically significant differences, particularly in the Game Implementation (t(38) = 6.37, *p* < .001, d = 0.67) and Overall Competency (t(38) = 6.94, *p* < .001, d = 0.70) sub-dimensions, indicate a medium to large practical significance, supporting H_1_ and H_2_. This suggests that the performance-oriented training of CE students provides a measurable advantage in game management and organizational skills.

### Qualitative findings: thematic analysis of tactical competency deficits

The thematic analysis of the semi-structured interviews with expert academics confirmed the quantitative findings and provided an in-depth contextual explanation, particularly regarding the pervasive deficiency in Tactical Competency. These themes emerged from expert narratives elicited through the structured prompts contained in the semi-structured interview guide (see Supplementary File 2, Interview Guide). The themes were classified under two main headings: Strengths (Organisation and Management) and Deficiencies in Tactical Intervention and Reflective Practice.

### Strengths

Experts consistently highlighted the high level of skill exhibited by candidates in the pre-game preparation and organizational processes. This finding is consistent with the highest average score in the quantitative Preparation Phase. Key themes included:

- *Organisation and planning competence: *Candidates demonstrated structured, systematic, and safe applications regarding material preparation, area arrangement, and rule explanation.
- *Leadership and classroom management skills: *Candidates effectively managed the class during the game process, exhibiting energetic, motivating, and guiding leadership.

### Deficiencies in tactical intervention and reflective practice

Under this heading, the experts pointed out critical deficiencies in the higher-order skills required for effective CCK, particularly those related to in-game tactical decision-making and post-game performance evaluation. These themes directly support the low scores observed in the Evaluation Phase and the need for a deeper understanding of the CE/PET difference.

#### Weakness in tactical intervention and stopping the game

Experts noted that candidates were insufficient in recognizing critical moments during the game and stopping the game for pedagogical reasons. Interventions were rarely used to create tactical awareness or have athletes review their decisions, indicating a lack of the skill to “turn the game into a teaching opportunity,” which is the fundamental principle of game-based models.

#### Lack of tactical teaching through questioning

A prominent theme was the candidates’ inadequacy in asking guiding questions aimed at in-team strategy, decision-making, and problem-solving. Candidates generally limited themselves to explaining “what they should do” rather than using inquisitive questions such as “why did you make such a decision?” or “would there have been a more effective solution?“. This deficiency limits the cognitive dimension of learning and decision-making development.

#### Insufficiency in reflective evaluation and feedback

Experts stated that candidates did not conduct evaluations with students on learning outcomes after the game and could not pedagogically conclude the process. Instead of providing structured feedback, applications were either passively terminated, or the gains were formally repeated. This finding is consistent with the Evaluation Phase sub-dimension receiving the lowest score in the quantitative analysis.

#### Lack of flexibility and adaptation in the game

Experts emphasized that candidates tended to implement the games as planned and did not engage in non-linear pedagogical interventions such as changing rules, restructuring roles, or differentiating according to class dynamics. This indicates that the skills to respond to varying athlete needs and “open different paths for learning” need to be developed.

### Integrated findings and general evaluation

A high level of consistency was observed between the quantitative and qualitative data. While the candidates’ strengths in preparation and organization areas stood out, post-game evaluation, tactical guidance, and questioning skills were determined as weak areas. Experts emphasized that the candidates’ skills in technically starting and managing the game were sufficient; however, they were open to development in terms of turning the game into a learning opportunity, increasing athletes’ cognitive participation, and evaluating the process. These findings indicate that pre-service educators need to be equipped not only with physical application skills but also with higher-order Tactical Competency skills such as tactical teaching, reflective evaluation, and intervention in in-game decision processes.

## Discussion

This study undertook a comprehensive and multilayered investigation into pre-service educators’ competencies in facilitating educational games, drawing on explanatory sequential mixed-methods data to illuminate the performance, cognitive, and organisational dimensions of game-based instruction. By integrating quantitative evaluations with qualitative insights from expert academics, the study was able to move beyond surface-level descriptions of technical performance and instead provide a theoretically grounded account of how CCK manifests or fails to manifest within practice-based instructional environments. The overarching aim was not only to determine existing competency levels but also to clarify the coaching mechanisms that underpin these competencies and identify systematic gaps that require curricular attention in the context of athlete development.

A central and recurrent finding across both data strands was the stark contrast between candidates’ strong organisational performance in the Preparation Phase and their comparatively weaker competencies in the Evaluation Phase. While preparation-related tasks such as material arrangement, safety considerations, and rule explanation were generally executed with proficiency, significantly lower scores emerged in areas requiring reflective, interpretive, and performance-outcome-oriented engagement. This pattern aligns with earlier national research^35,41^ and echoes international findings^11,42^, which collectively reveal persistent global challenges in coaches’ ability to integrate reflective evaluation into game-based instruction. Such limitations are not trivial; they point directly to underdeveloped components of CCK, especially those related to performance assessment literacy, athlete decision-making understanding, and instructional decision-making, which are core to effective coaching^43^.

The quantitative superiority of CE candidates in game management and organizational dimensions (H₁ and H₂) suggests a more robust functional socialization within their specific training pathway. This finding can be critically analyzed through the lens of Occupational Socialization Theory, which posits that the “apprenticeship of observation” and the specialized nature of coach training programs shape the practitioner’s ability to navigate complex instructional environments^26,33^. In the Turkish context, the CE curriculum’s emphasis on “Specialization Courses” provides a sustained, performance-oriented immersion that PET candidates often lack^31^. This intensive exposure likely fosters a higher degree of Pedagogical Content Knowledge, specifically related to game flow and organizational efficiency. As noted by Pill et al.^23^, practitioners who are socialized in environments that prioritize tactical performance and athlete development are more likely to internalize the logistical demands of game-based approaches (GBAs). The CE candidates’ ability to manage the transition from preparation to implementation more effectively than their PET counterparts reflects a successful integration of theoretical LTAD models into functional practice. However, this organizational proficiency should not be mistaken for complete pedagogical mastery, as it primarily addresses the “surface-level” management of the game rather than its deep tactical facilitation. This distinction is critical; the ultimate goal of GBAs is not merely to facilitate physical activity, but to cultivate deep tactical understanding and decision-making skills that support athletes’ cognitive, affective, and social development^6,7^. An overly directive and performance-focused socialization may inadvertently constrain coaches from adopting more athlete-cantered or reflective approaches, thereby limiting the full potential of GBAs^16^. This highlights a potential disconnect between “what is taught” and “how it is taught” in CE; while organizational skills are often readily observable and assessable, the development of more complex pedagogical skills requires more structured experience and reflection^43,44^. This finding, while demonstrating the strengths of the CE curriculum, also provides a roadmap for the next stage of pedagogical evolution: moving beyond organizational efficiency to integrate more nuanced instructional strategies that foster athlete autonomy and critical thinking^10,14^.

Despite the organizational advantages observed in CE candidates, the qualitative data revealed a pervasive and shared deficiency in higher-order tactical intervention and reflective evaluation across both cohorts (H₃). This “plateau” in competency development represents a significant Theory-Practice Gap that is well-documented in pre-service education literature^24^. Critically, the inability of both PET and CE candidates to provide nuanced, in-game tactical facilitation suggests a limitation in their CCK. From a Cognitive Load Theory perspective, pre-service practitioners often experience high intrinsic load when managing the logistical and technical aspects of a game, which leaves insufficient germane resources for the complex task of real-time tactical analysis and reflective feedback^45,46^. The expert academics’ observations that candidates remained “descriptive” rather than “interpretive” in their evaluations further underscore this point. This suggests that while CE students are better equipped to “run” a game, they are not necessarily better equipped to “teach” the game at a high tactical level. This shared deficit points to a structural weakness in both curricula: an over-reliance on technical and organizational training at the expense of developing the sophisticated, reflective, and evaluative expertise required for authentic game-based pedagogy. Without explicit, practice-based modules that target these high-level cognitive skills, pre-service educators are likely to revert to traditional, command-style instruction when faced with the fluid demands of tactical facilitation. This situation contradicts modern pedagogical expectations, particularly in sports science, which dictate that coaches must effectively manage not only the technical and physical dimensions of the game but also the cognitive and decision-making processes^5,9,47^. In this context, it is critical that the curriculum is enriched with practical experiences, such as simulations and case studies, aimed at developing coaches’ rapid and effective decision-making skills in complex game situations^48^. Furthermore, this deficiency may prevent coaches from transcending the role of mere practitioners who transmit technical skills, hindering their ability to act as facilitators who develop athletes’ autonomous decision-making and problem-solving capabilities^10,16^. This significantly disrupts the effective implementation of athlete-centered learning approaches, a core principle of modern sports pedagogy^14^. These findings demonstrate that CE must focus not only on “what” is taught but also on “how” it is taught, highlighting the necessity of revising pedagogical strategies specifically to develop complex tactical thinking and reflective practice skills^13,49^. This in-depth analysis clearly reveals the ongoing challenges in translating theoretical knowledge into practical application in pre-service CE, particularly in areas such as tactical intelligence and reflective pedagogy. It indicates a need for innovative approaches in training programs that allow coaches to develop a “pedagogical flexibility” capable of supporting not only the mechanical aspects of the game but also the cognitive and emotional development of athletes^27,50,51^.

The present findings confirm that while pre-service educators can manage the logistical and procedural aspects of educational games, they often struggle to transform these games into meaningful skill acquisition and performance optimization opportunities^52^. This difficulty becomes particularly evident in areas requiring coaches to pause games strategically, pose cognitively demanding questions, or guide athletes in making sense of tactical decisions, skills central to contemporary coaching models such as TGfU and Game Sense^16,47^. According to CCK-specific frameworks in sports coaching, these skills correspond to higher-order domains of coaching knowledge, including the ability to diagnose performance needs, connect game actions with conceptual principles, and adapt tasks based on athletes’ responses^19^. The findings thus suggest that candidates possess foundational CCK related to organisation and management but lack the more sophisticated, Tactical Competency dimensions necessary for high-impact, performance-focused game-based instruction.

The qualitative findings provided further clarity by demonstrating that, although candidates excelled in technical tasks such as explaining rules, managing space, and maintaining discipline, they exhibited notable deficits in Tactical Competency elements: performance reflection, tactical questioning, and post-game performance analysis. Expert observers consistently noted that candidates rarely used open-ended questions to stimulate athlete decision-making, seldom paused games to address performance or tactical patterns, and frequently ended sessions without linking game events to intended performance outcomes. This aligns with concerns raised in the literature^9,53^, which argue that practitioners often treat game-based lessons as opportunities for physical engagement rather than cognitive and tactical development. Without deliberate reflection, the performance value of game-based learning is significantly diminished^49^.

The limited capacity for spontaneous adaptation observed in many candidates further suggests insufficient training in non-linear pedagogy. Educational games require dynamic and situational decision-making skills that extend beyond pre-scripted lesson plans. Yet many candidates adhered rigidly to predetermined structures, demonstrating difficulty in modifying rules, differentiating tasks, or redesigning flow based on emergent athlete needs. This tendency conflicts with the central tenets of ecological and constraints-led approaches^9^, which are fundamental to modern skill acquisition literature and position learning as an adaptive process requiring flexible instructional responsiveness^47^.

### Addressing previous criticisms

The present study addresses key limitations identified in previous research and provides a robust response to the criticisms raised during the initial submission process:

- Novelty and originality: This study offers significant novelty by being one of the first to systematically measure Coaching Competency specifically the high-level cognitive skills of tactic decision-making and reflective evaluation among pre-service coaches, moving beyond traditional assessments of motor skills or technical knowledge. By comparing CE and PET cohorts, we isolate the structural deficiencies in Tactical Competency that persist even within performance-focused training programs, a finding with direct implications for the effectiveness of LTAD implementation.
- Methodological rigour: The use of an Explanatory Sequential Mixed Methods design is not merely a descriptive choice but a methodological necessity. Coaching competency is a multi-dimensional construct that cannot be fully captured by quantitative metrics alone. The mixed-methods approach was essential to: (a) quantify the observable performance deficits (quantitative phase) and (b) provide the necessary conceptual insight and interpretive depth (qualitative phase) to explain why these deficits exist, linking them to structural gaps in Coaching Content Knowledge development. This design ensures the rigour required to capture the complexity of real-time coaching behaviour.

These findings collectively point toward the need for more robust integration of applied CCK development within both PET and CE curricula. Specifically, programmes should ensure systematic opportunities to practice and refine the reflective, tactical, and evaluative dimensions of game-based coaching. This could involve structured video analysis tasks, simulation-based coaching experiences, or targeted micro-teaching modules where students receive immediate feedback on their tactical questioning and in-game decision-making processes. The incorporation of a dedicated course such as “Tactical Intervention and Performance Evaluation,” as proposed by the present study, would enable students to internalise the epistemological and coaching foundations of game-based models and practice the higher-order instructional competencies they require.

Moreover, alternative and authentic assessment strategies such as athlete-focused reflections, peer evaluations, digital portfolios, and game performance assessments would allow future coaches to better understand how their instructional decisions shape performance outcomes. Such approaches align with current international recommendations for competency-based CE, which emphasise reflective practice and inquiry-oriented coaching.

## Conclusion

This research provides a strong empirical basis that the educational game playing competencies of pre-service coaches suffer from a significant, shared deficit in Tactical Competency that extends beyond mere technical proficiencies. The comparative analysis with PET students, while showing CE students’ superior organizational skills, highlights that the common deficiency in reflective and evaluative practices represents a structural gap in the development of CCK within performance-focused training programs^44,48^.

The findings transcend the local context, necessitating a global re-evaluation of how CCK is currently integrated into CE curricula worldwide. Specifically, the observed inability of pre-service coaches to execute timely tactical interventions and conduct meaningful post-game performance evaluations directly compromises the effectiveness of modern athlete development models, such as LTAD^12^ and DSPM^13^. This deficiency means that future coaches are ill-equipped to foster the high-level decision-making skills and game intelligence required for optimal athlete performance and long-term engagement in sport.

These findings suggest the need to reconsider how tactical facilitation and evaluative competencies are systematically integrated within CE curricula. Greater emphasis on structured, practice-based modules focusing on tactical intervention and performance evaluation may strengthen the quality of game-based implementation. Rather than demonstrating causal impact, the findings highlight facilitation patterns that may condition the effectiveness of game-based implementation.

### Limitations and future research

While the study presents strong empirical evidence, its limitations warrant consideration. The focus on a single institution and a relatively small expert panel may limit generalisability. Future research should adopt multi-institutional and cross-cultural designs, longitudinal approaches to competency development, and experimental interventions comparing different pedagogical training models. Such research could further refine our understanding of how CCK develops in game-based instruction and what curricular structures best support this development.

### Impact statement

This study provides novel, mixed-methods evidence of a critical, shared deficit in Tactical Competency (a core component of Coaching Content Knowledge) among pre-service coaches, specifically concerning in-game tactical intervention and performance evaluation. The findings offer strong empirical justification for international CE bodies to urgently reform curricula, prioritizing the explicit development of these high-level cognitive skills to ensure future coaches can effectively implement performance-enhancing models like LTAD and optimize athlete decision-making and long-term development.

## Supplementary Information

Below is the link to the electronic supplementary material.

Supplementary Material 1

## Data Availability

The authors E.A and Y.A. confirms that the data that support the findings of this study are openly available in the following link: [https://www.scidb.cn/en/anonymous/RjdWRkZm](https:/www.scidb.cn/en/anonymous/RjdWRkZm).

## Abbreviations

CCK: Coaching content knowledge
CE: Coach education
CI: Confidence interval
DSPM: Developmental sport participation model
GBAs: Game-based approaches
LTAD: Long-term athletic development
M: Mean
OST: Occupational socialization theory
PET: Physical education teacher
SD: Standard deviation
TGfU: Teaching games for understanding
