# Supplementary material for: Tactical and assessment competency gaps in pre-service coaches: evidence from a game-based implementation
Source: Sci Rep. 2026 May 24;16:23736. doi: 10.1038/s41598-026-53681-3 (PMC13429655; doi:10.1038/s41598-026-53681-3)
Supplement: Supplementary file 1 — Supplementary Material 1 [file 41598_2026_53681_MOESM1_ESM.docx]

**Supplementary File 2**

**Semi-Structured Interview Form (English Version)**

**Description**

This semi-structured interview form was developed specifically for the present study by two researchers (Acar & Akinci) in order to obtain in-depth qualitative data regarding pre-service coaches’ competencies in planning, implementing, and evaluating educational games. The interview questions were designed in alignment with the quantitative assessment framework and were structured around three main phases of the educational game process: preparation, implementation, and evaluation.

The form aims to explore expert perspectives on candidates’ organizational, instructional, and reflective competencies within the context of educational game-based instruction.

**Ethical Considerations**

Participation in the interviews was voluntary. Prior to data collection, all participants were informed about the purpose of the study, the confidentiality of their responses, and their right to withdraw from the interview at any stage without penalty. Informed consent was obtained from all participants. Audio recordings were used solely for research purposes and were transcribed verbatim. All data were anonymized to ensure participant confidentiality and were handled in accordance with ethical research guidelines.

**Semi-Structured Interview Questions**

**Section 1:** Preparation Phase of the Educational Game

*(Organizational CCK – Content Knowledge and Context Knowledge)*

1-How would you evaluate the candidate’s preparation phase of the educational game in terms of selecting and organizing the game area and materials? In your opinion, to what extent were the game environment, materials, and designated player areas (e.g., teams, eliminated players, waiting zones) appropriate for the game’s objective, participants’ level, and safety requirements?

2-What do you think are the most critical considerations during the preparation phase of an educational game, and how successfully did the candidate address these aspects?
Please comment on strengths and possible shortcomings related to material suitability, practicality, clarity, and safety precautions.

**Section 2:** Game Implementation Phase

*(Tactical Intervention CCK – Instructional Strategies Knowledge)*

1-How clearly and effectively did the candidate explain the rules, roles, and flow of the game to the participants? Please evaluate the explanation of rules, role distribution, use of demonstrations, and confirmation of whether the players fully understood how to play the game.

2-How would you assess the candidate’s ability to manage the game process and make timely instructional interventions? In your view, how effectively did the candidate ensure participation, maintain discipline, balance the game, respond to unexpected situations, and adapt the game when necessary?

**Section 3:** Evaluation Phase of the Educational Game

*(Reflective CCK – Knowledge of Learning Outcomes and Assessment)*

1-How did the candidate conduct the evaluation process at the end of the game in relation to the intended learning outcomes? To what extent were feedback, reinforcement, rewards or penalties, and time management aligned with the educational purpose of the game? In your opinion, how effectively did the candidate link the game outcomes to the targeted learning gains?
2-Please explain whether the candidate was able to guide reflection, reinforce key learning points, and ensure that the evaluation process supported meaningful learning.
